# L-Type amino acid transporter 1 as a target for drug delivery

**DOI:** 10.1007/s11095-020-02826-8

**Published:** 2020-05-06

**Authors:** Elena Puris, Mikko Gynther, Seppo Auriola, Kristiina M. Huttunen

**Affiliations:** 1 School of Pharmacy, University of Eastern Finland, P.O. Box 1627, FI-70211 Kuopio, Finland; 2 Institute of Pharmacy and Molecular Biotechnology, Ruprecht-Karls-University, 69120 Heidelberg, Germany

**Keywords:** drug delivery systems, L-type amino acid transporter 1, membrane transporter, targeting

## Abstract

Our growing understanding of membrane transporters and their substrate specificity has opened a new avenue in the field of targeted drug delivery. The L-type amino acid transporter 1 (LAT1) has been one of the most extensively investigated transporters for delivering drugs across biological barriers. The transporter is predominantly expressed in cerebral cortex, blood-brain barrier, blood-retina barrier, testis, placenta, bone marrow and several types of cancer. Its physiological function is to mediate Na^+^ and pH independent exchange of essential amino acids: leucine, phenylalanine, etc. Several drugs and prodrugs designed as LAT1 substrates have been developed to improve targeted delivery into the brain and cancer cells. Thus, the anti-parkinsonian drug, L-Dopa, the anti-cancer drug, melphalan and the anti-epileptic drug gabapentin, all used in clinical practice, utilize LAT1 to reach their target site. These examples provide supporting evidence for the utility of the LAT1-mediated targeted delivery of the (pro)drug. This review comprehensively summarizes recent advances in LAT1-mediated targeted drug delivery. In addition, the use of LAT1 is critically evaluated and limitations of the approach are discussed.

## Introduction

The main aim of targeted drug delivery is to achieve therapeutic concentrations of the drug at the site of action in the tissue of interest to produce the desired pharmacological effect as well as minimizing the side effects caused by drug distribution to other organs. Delivery of the drug to the site where the target is located requires knowledge about the tissue and an understanding of what features the drug should possess in order to be selectively distributed. In addition to direct administration of the drug by some specific route to the target organ, several other strategies have been investigated including nanoparticle-based drug delivery systems, prodrugs or derivatives and stimuli sensitive targeted therapy (1,2).

One of the most promising approaches is the utilization of the transporters selectively expressed at the cell membrane of the target tissue (3,4). The design of drugs or prodrugs as substrates of the particular membrane transporter can enable targeted delivery of the drug based on the tissue specific expression profile of the carrier. In this respect, the L-type amino acid transporter 1 (LAT1, SLC7A5), which is expressed at a relatively high level at the blood-brain barrier (BBB), blood-retinal barrier (BRB), testis, bone marrow, placenta and several types of human tumour is an intriguing way for targeting a drug to these organs. Importantly, the transporter has already demonstrated its utility by mediating the brain delivery of the antiparkinsonian drug L-Dopa and the anticancer drug, melphalan (5,6). This suggests that effective drug delivery to the target organ expressing LAT1 can be achieved by rational design of the drugs mimicking the structures of the transporter endogenous substrates.

In the present review, the current state of the art and recent improvements in the LAT1-mediated drug delivery are summarized. In addition, the limitations and advantages of the approach are discussed.

## LAT1 and its function

**Fig. 1:**
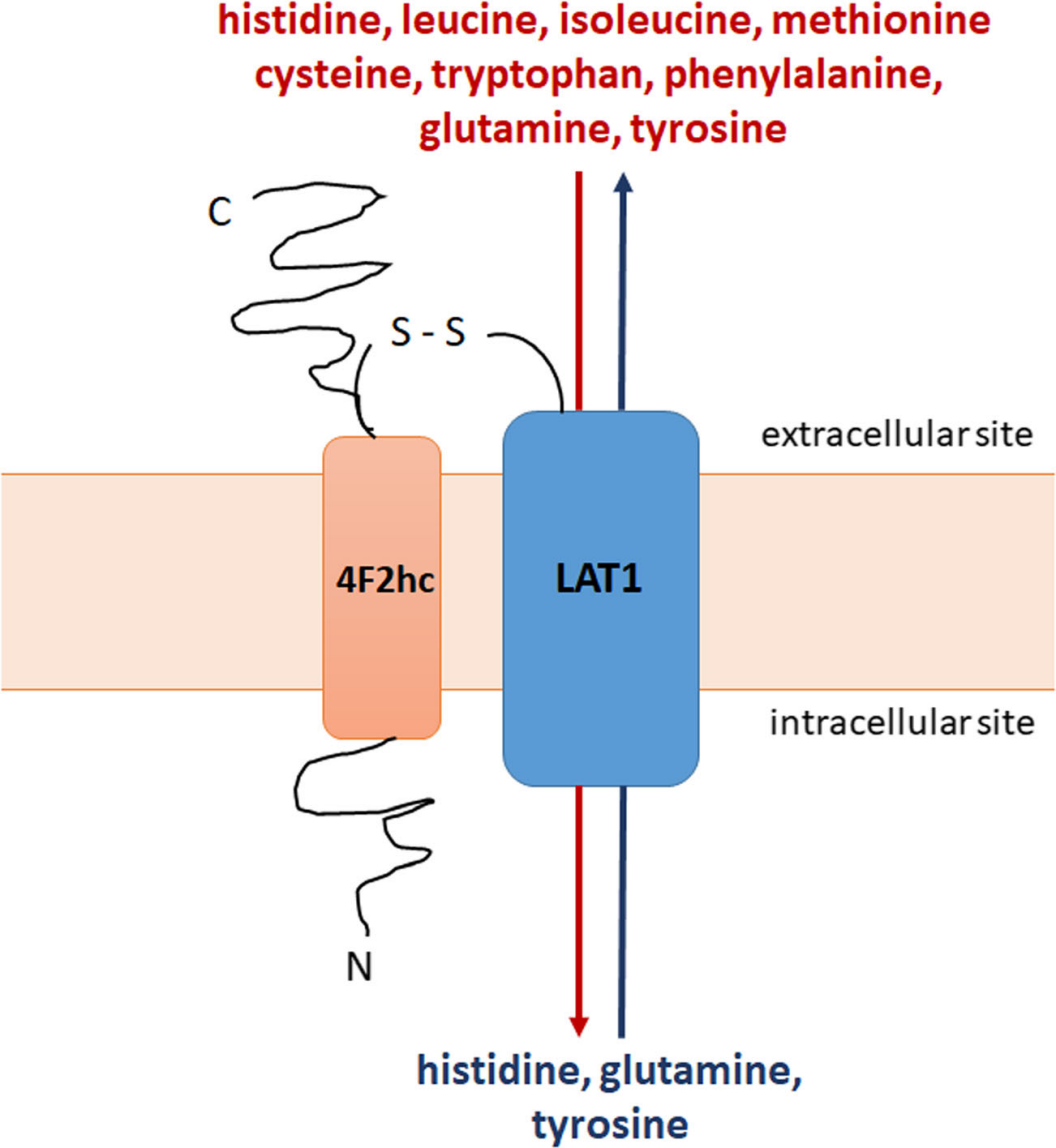
Heterodimeric transporter LAT1/4F2hc - mediated transport of essential (histidine, isoleucine, methionine, tryptophan, phenylalanine, leucine, cysteine, tyrosine) and non-essential (glutamine) amino acids across the cell membrane.

The L-type amino acid transporter 1 is a Na^+^- and pH-independent exchanger of large branched-chain and aromatic neutral amino acids with 1:1 stoichiometry (7,8). The transporter consists of two subunits covalently linked via a disulfide bond (9–11). The light chain subunit (LAT1, SLC7A5) is a functional subunit responsible for the exchange of amino acids (Fig. 1). The heavy chain subunit (known as CD98 or 4F2hc, SLC3A2) is a glycoprotein coupled with the light chain subunit which acts as a molecular chaperone localizing LAT1 at the plasma membrane. In addition, it is involved in the processes of cell survival and integrin activation. Recently, a cryo-electron microscopy study investigating the structure of the complex LAT1-4F2hc (hereinafter referred to as LAT1) revealed that in addition to a disulfide bond association, the light chain subunit cooperates extensively with 4F2hc on the extra- and intracellular side of the membrane as well as within the membrane (12). The authors concluded that the heavy chain subunit was crucial for the transport function of the complex (12).

The transporter is responsible for the inward flux of several essential amino acids such as phenylalanine, leucine, isoleucine, tryptophan, histidine, tyrosine in antiport with histidine, tyrosine and a non-essential amino acid glutamine (Fig. 1) (8,13–15). While the affinity of the essential amino acids for LAT1 is high (*K*_m_ values for the human isoform range from 14 to 28 μM) (9,13,16), the non-essential glutamine showed lower affinity (*K*_m_ 1.6 mM) to human LAT1. The intracellular accumulation of glutamine acting as an exchanger for essential amino acids is mediated by the transporters of system N and A (13,16,17). In addition to amino acid transport, LAT1 plays a role in the passage of thyroid hormones, i.e. triiodothyronine and thyroxine (18).

The LAT1 plays a crucial role in normal body functioning by regulating the exchange of the amino acids which take part in the synthesis of peptides, proteins and neurotransmitters as well as in nutrient metabolism. Thus, while developing LAT1-mediated drug delivery systems for targeting non-tumorous tissue, it is important that the vital function of the transporter should not be disturbed. In contrast, as the transporter plays a crucial role in cancer cell growth and survival, a strategy to affect the function and expression of LAT1 in a tumour has been investigated and considered as a promising treatment approach (19). More information regarding the structure, function, transport mechanism and homology modelling of LAT1 can be found in recently published studies and reviews (14,20–26).

## Tissue expression of LAT1

The localization and mRNA/protein expression of the light chain subunit LAT1 in specific human tissues according to the Human Protein Atlas (27) is summarized in Table I. The mRNA expression of the LAT1 differs in the 27 studied human tissues with the highest expression present in the cerebral cortex, retina, esophagus, testis, placenta and bone marrow (27). LAT1 is primarily localized in the basolateral membrane of polarized epithelia (28,29). The highest levels of LAT1 protein expression of LAT1 were detected in parathyroid and adrenal glands, salivary gland, esophagus, bronchus, kidney, urinary bladder, bone marrow, appendix, testis, heart muscle, fallopian tube, cervix, gastrointestinal tract, smooth muscle and gallbladder (Table I).

**Table I:** The Localization and mRNA/Protein Expression of LAT1 in Human Tissues According to the Human Protein Atlas (27)

**Tissue**	mRNA*	**Protein**
Cerebral cortex including endothelial cells and neurons, cerebellum	15.9–45.8	medium
Olfactory region, hippocampus, amygdala, thalamus, hypothalamus, midbrain, pons and medulla, corpus callosum, spinal cord	9.6–24.5	N/A
Basal ganglia	24.5	low
Retina	34.9	N/A
Thyroid gland	4.2	medium
Parathyroid and adrenal glands	1.5–3.2	high
Pituitary gland	9.0	N/A
Salivary gland and esophagus	11.5–38.7	high
Oral mucosa	N/A	medium
Tongue	16.7	N/A
Nasopharynx	N/A	medium
Bronchus	N/A	high
Lung	3.8	medium
Kidney and urinary bladder	3.6–11.5	high
Gastrointestinal tract: stomach, duodenum, small intestine, colon, rectum	3.4–12.7	high
Epididymis, seminal vesicle and prostate	4.3–30.4	medium
Testis		high
Ovary, endometrium, placenta and breast	4.3–28.4	medium
Fallopian tube and cervix	3.3–7.9	high
Heart muscle	8.2	high
Skeletal muscle	11.2	medium
Smooth muscle	4.8	high
Adipose tissue	8.2	N/A
Liver and gallblader	9.7	medium
Gallblader	6.4	high
Skin	10.7	medium
Lymph node	11.1	low
Spleen and tonsil	6.4–15.0	medium
Bone marrow	71.2	high
Thymus	3.7	N/A
Appendix	14.7	high
Blood cells including granulocytes, T- and B-cells, NK cells, dendritic cells	0.2–15.9	N/A

N/A – no data available

*- normalized expression levels.

In the endothelial cells of the brain microvessels, LAT1 is expressed on both apical and basolateral sides of the cell membranes (30). Additionally, LAT1 expression was detected in brain parenchymal cells such as human and mouse astrocytes, mouse and rat neurons and immortalized microglia cultures (31–33). LAT1 is also expressed in the inner BRB, ensuring the flux of large neutral amino acids and neurotransmitters (34).

Furthermore, LAT1 is overexpressed in human tumors such as cholangiocarcinoma, malignant glioma, multiple myeloma, and lung, bladder, bone, pancreas, thyroid, prostate, uterine cervical, breast cancer and other malignancies as compared to benign tissue used as the control (9,16,35–37). The association between LAT1 overexpression and meaningfully shorter survival in many types of cancer have indicated that the transporter may be exploited as a prognostic biomarker to predict the outcome in different cancer types (38,39).

The protein expression of LAT1 in the majority of the listed tissues has been also confirmed, although quantitative information about the absolute protein expression is still limited. Importantly, the protein expression of LAT1 can vary between species, which should be taken into account during the development of LAT1-utilizing (pro)drugs. For instance, in isolated cortex microvessels, the protein expression in rats (3.00 ± 0.62 fmol/μg protein) and mice (2.19 ± 0.21 fmol/μg protein) was higher than in humans (0.43 ± 0.09 fmol/μg protein) (40,41).

There is a correlation between the mRNA expressions of the heavy chain SLC3A2 and light chain SLC7A5 in human tissues. Similarly to SLC7A5, the highest levels of SLC3A2 mRNA expression were found in cerebral cortex, placenta, testis and bone marrow (27). This correlation hints at the presence of an interaction between the subunits. Additionally, high SLC3A2 expression has been detected in other tissues, i.e. parathyroid gland and kidney, where the heavy chain is responsible for localizing other SLC7 transporters at the cell membrane (27,29).

## LAT1 and diseases

The expression and function of LAT1 can be altered in pathological conditions, such as cancer and central nervous system (CNS) diseases and consequently, this can lead to altered efficacy of LAT1-mediated drug delivery to the target organ.

Thus, overexpression of LAT1 in human brain metastasis in comparison to non-tumoral brains correlated to increased uptake of LAT1 substrate 6-[^18^F]-fluoro-L-3,4-dihydroxy-phenylalanine ([^18^F]-DOPA) (33). In the 1-methyl-4-phenyl-1,2,3,6-tetrahydropyridine treated mouse model of Parkinson’s disease, mRNA expression of LAT1 at the BBB was significantly reduced, although this was not reflected in the protein expression level of the transporter (42). In addition, the loss of LAT1 due to mutations leads to impairment of amino acid homeostasis in the brain and therefore it has been suggested to be a cause of motor dysfunction and Autism Spectrum Disorder in mice and human (43). Recently, Gynther et al. (2018) showed that LAT1 function was not altered at the BBB of lipopolysaccharide (LPS) induced neuroinflammation murine model and in the transgenic Alzheimer’s disease model with amyloid precursor protein (*APP*) and presenilin (*PSEN1*) gene mutations, (31). In the same study, LAT1 protein expression and function were similar in wild type astrocytes with and without LPS treatment and in *APP/PS1* transgenic astrocytes treated with LPS (31).

Furthermore, as previously mentioned due to upregulated LAT1 expression in several human cancers and its role in tumour cell growth and survival, the transporter has been considered as the potential target for anticancer therapy. The LAT1 inhibition as a strategy for cancer treatment has been summarized in several excellent reviews (14,19–21,44–47). In addition, Cibrian et al. (2020) demonstrated that LAT1 expression was upregulated in keratinocytes and skin infiltrating lymphocytes of psoriatic lesions in human subjects and mice (48). The authors considered targeting LAT1 as a potential immunosuppressive strategy to regulate skin inflammation driven by the interleukin IL-23/IL-1b/IL-17 axis (48).

## Drug delivery via LAT1

### Substrate specificity

One obstacle to the development of LAT1-utilizing compounds can be the potential inhibition of their uptake due to competition with amino acids. The essential amino acids are delivered into the brain mainly from the blood after dietary intake, while non-essential amino acids are synthetized inside the brain. Thus, the food or supplements containing high amounts of essential amino acids or protein can alter the delivery of LAT1 utilizing (pro)drugs. For instance, the brain uptake of L-Dopa, a substrate of LAT1, was decreased after a high-protein meal or the intravenous infusion of large neutral amino acids before the administration of the drug in 1-methyl-4-phenyl-1,2,3,6-tetrahydropyridine parkinsonian monkeys (49). Nonetheless, the pilot study of Cucca et al. (2015) demonstrated that a six-month amino acid supplementation in protein-restricted patients with Parkinson’s disease, chronically treated with L-Dopa, did not affect neurological parameters (50).

The efficient utilization of LAT1 for drug delivery requires the rational design and development of compounds which can consequently compete with millimolar concentrations of amino acids. One of the strategies to overcome this issue is to develop (pro) drugs with higher affinity (*K*_*m*_) to LAT1 as compared to that of the endogenous amino acid substrates. In this respect, it is important to gather knowledge about substrate specificity and an understanding of ligand - transporter interactions. Despite the relatively low maximal transport rate (*V*_*max*_) enabling the substrate compounds to cross the cellular membrane per unit time, the amino acid-resembling (pro) drugs have demonstrated a potential to utilize the transporter for CNS and cancer delivery.

Several studies investigating the development of LAT1 substrate structure-activity relationship have been conducted (23,51–55). The findings suggest that binding to LAT1 requires the presence of both the amino and carboxylic acid functional groups as well as large, neutral side groups. In addition, the presence of aromaticity in the compound molecule, for instance in a prodrug promoiety such as phenylalanine, plays an important role in the binding to LAT1 and its ability to utilize transporter for cellular uptake (53,56,57).

Importantly, it has been demonstrated that the affinity (*K*_m_) to LAT1 of L-enantiomers of phenylalanine and leucine was higher as compared to the corresponding D-enantiomers. In contrast, the transport rate (*V*_max_) was similar for L and D-enantiomers (16). In the study of Chien et al. (2018), the authors concluded that LAT1 is not stereoselective in terms of the transport rate of the amino acids, whereas based on the relative IC_50_-values of L- and D-enantiomers of amino acid, there is variation in their binding to the transporter (58).

In addition, LAT1 has overlapping substrate specificity with other amino acid transporters, for instance LAT2 (20), which can complicate the development of LAT1 selective compounds for the targeted delivery.

### Brain delivery of CNS-acting drugs

**Fig. 2:**
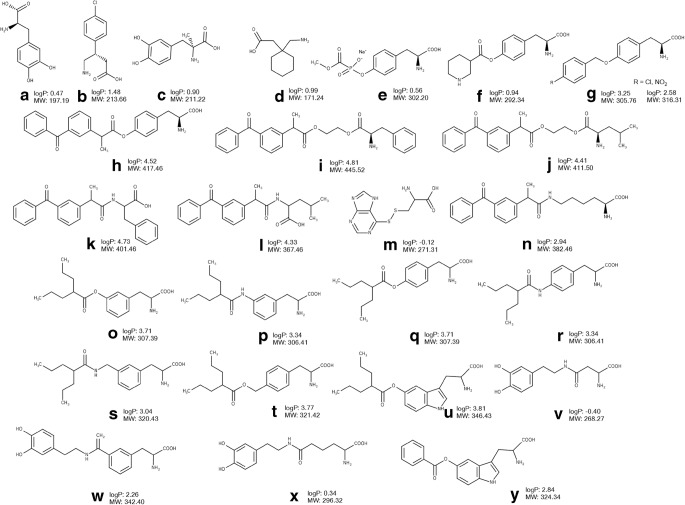
Chemical structures, molecular weight (MW) and logP of amino acid CNS drugs and derivatives designed to utilize LAT1 (part 1): L-Dopa (**a**), baclofen (**b**), alpha-methyldopa (**c**) gabapentin (**d**), derivative of phosphonoformate (**e**), derivative of nipecotic acid (**f**), derivative of L-tyrosine (**g**), derivatives of ketoprofen (**h**-**l**, **n**), derivative of 6-mercaptopurine (**m**), derivatives of valproic acid (**o**-**u**), derivatives of dopamine (**v**-**x**), derivative of benzoic acid (**y**). The values of logP were calculated using Marvin Sketch version 15.8.31 (ChemAxon, Budapest, Hungary)0

The comparatively high expression of LAT1 transporter on both the luminal and abluminal sides of the brain capillary endothelial cells as well as on brain parenchymal cells offers a promising opportunity for LAT1-mediated brain delivery of CNS drugs. The transporter has demonstrated its efficacy in the delivery of clinically used CNS drugs and prodrugs such as L-Dopa (Fig. 2A), baclofen (Fig. 2B), alpha-methyldopa (Fig. 2C) gabapentin (Fig. 2D) (5,6,59,60).

**Fig. 3:**
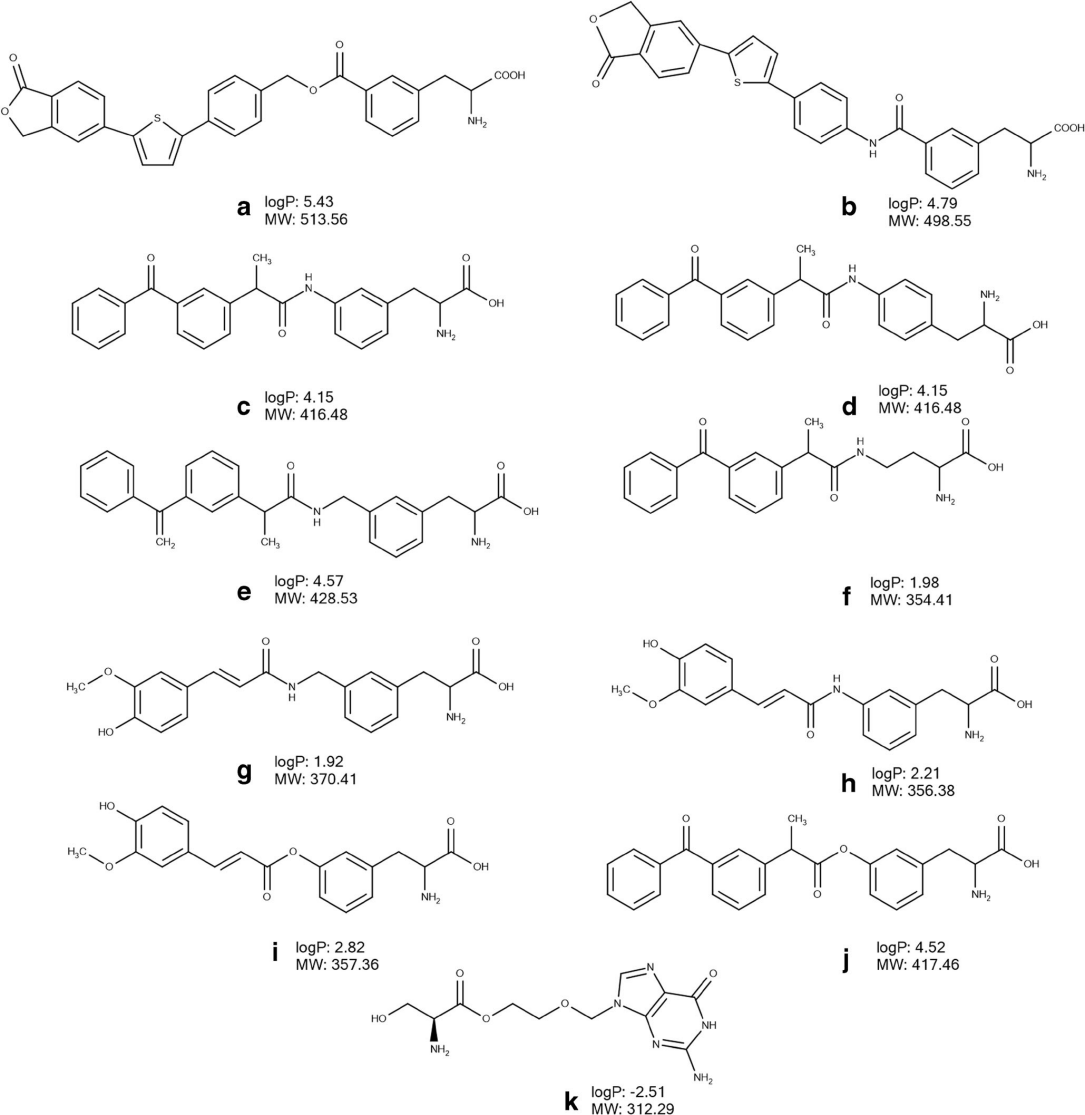
Chemical structures, molecular weight (MW) and logP of amino acid CNS drugs and derivatives designed to utilize LAT1 (part 2): derivatives of perforin inhibitor (**a**, **b**), derivatives of ketoprofen (**c**-**f**, **j**), derivatives of ferulic acid (**g**, **i**), derivative of acyclovir (**k**). The values of logP were calculated using Marvin Sketch version 15.8.31 (ChemAxon, Budapest, Hungary).

Several attempts have been made to develop LAT1-utilizing derivatives and prodrugs sharing the structural features of the natural substrates. Some derivatives have demonstrated efficient LAT1-mediated cellular uptake and/or BBB permeation after *in situ* brain perfusion in rats and mice (61–66). In the majority of cases, a parent drug has been conjugated to the amino acid side chain via a biodegradable linker in such a way that carboxyl and amino groups are not substituted to allow effective LAT1 binding (Figs. 2, 3).

Thus, the L-tyrosine derivative of antiviral phosphonoformate (Fig. 2E) synthetized by Walker et al. (1994) inhibited the uptake L-[^3^H]-tyrosine in porcine brain microvessel endothelial cells (67) providing evidence of LAT1 binding. A tyrosine conjugate of another CNS agent, nipecotic acid (Fig. 2F), designed as a substrate of the amino acid transporter displayed a concentration-dependent anticonvulsant effect in a murine epilepsy model - Diluted Brown Agouti/2 mice (68). In another study, *p*-nitro- and *p*-chlorobenzyl ether prodrugs of L-tyrosine (Fig. 2G) inhibited the uptake of L-[^3^H]-tyrosine in rabbit corneal cells (69). Gynther et al. (2008) demonstrated LAT1-mediated delivery of an L-tyrosine prodrug of ketoprofen (Fig. 2H) across the BBB using the *in situ* rat brain perfusion technique, as the brain uptake of the prodrug was significantly decreased after co-perfusion with the competitive LAT1 inhibitor, 2-amino-2-norbornanecarboxylic acid (BCH) (62). The ester- or amide-based ketoprofen prodrugs (Fig. 2I-L) conjugated to either phenylalanine or leucine did not show any binding to LAT1, as the brain uptake of L-[^14^C]-leucine was not altered after co-perfusion with these prodrugs in *in situ* brain perfusion in rats (62). This provides additional evidence that the presence of α-carboxyl and α-amino groups as well as a nonpolar side chain in the structure of substrate of the transporter is important for binding to LAT1. Killian et al. (2007) covalently conjugated L-cysteine via a disulphide bond to 6-mercaptopurine (Fig. 2M) and showed that the prodrug significantly inhibited the uptake of L-[^14^C]-leucine across the BBB after *in situ* brain perfusion in rats, thereby demonstrating affinity to LAT1 (63).

The neuropharmacokinetic study of the LAT1-utilizing L-lysine derivative of ketoprofen (Fig. 2N) using a cerebral microdialysis technique in rats revealed for the first time that after crossing the BBB, the prodrug was predominantly distributed into the brain intracellular compartment and subsequently this was followed by the release of the parent drug (61). Thus, the LAT1-mediated uptake of the prodrug was supported by the inhibition of the brain uptake of L-[^14^C]-leucine after *in situ* perfusion in rats, causing a reduction in the brain uptake of prodrug after co-perfusion with a LAT1 substrate, L-phenylalanine (61). The ratio of unbound parent drug AUC in brain extracellular fluid to plasma was more than doubled after prodrug dosing as compared to ketoprofen itself given as an i.v. bolus injection in rats (61). In addition, the estimated brain intracellular delivery efficacy evaluated as the AUC ratio of unbound parent drug in the brain intracellular compartment to plasma was significantly higher after prodrug dosing (21.8) in comparison to parent drug administration (0.06) (61). In another study, Peura et al. (2011) synthetized several LAT1-utilizing prodrugs of valproic acid and showed that *meta-*substituted amide- and ester-based phenylalanine derivatives (Fig. 2O, P) had 10-fold higher affinity to LAT1 in comparison with the *para*-substituted conjugates as demonstrated by inhibition of L-[^14^C]-leucine brain uptake by prodrugs after *in situ* brain perfusion in rats (Fig. 2Q, R) (65). Importantly, the amide-based *meta*-substituted prodrug (Fig. 2P) displayed 2-fold greater permeation rate across the BBB as compared to the *para*-substituted prodrug of valproic acid (Fig. 2R) as evaluated with *in situ* brain perfusion in rats (65). Furthermore, the ability to bind to LAT1 was investigated for these prodrugs (Fig. 2O-R) as well as for amide- and ester-based prodrugs of valproic acid (Fig. 2S,T) conjugated to phenylalanine with an additional methylene group and tryptophan prodrug of valproic acid (Fig. 2U) (70). The study demonstrated that the prodrugs inhibited the uptake of the LAT1 substrate L-[^14^C]-leucine in human breast cancer MCF-7 cells i.e. evidence of binding to LAT1. In addition, in pharmacokinetic study in rats, after a single i.v. bolus injection, amide-based prodrugs of valproic acid (Fig. 2P,R,S) crossed the BBB and released the parent drug, although the extent of brain delivery of valproic acid was not improved (70). Thus, the brain AUC and C_max_ of the parent drug released after dosing with the prodrugs was more than 7 times lower than those after valproic acid administration (70). In another study, Peura et al. (2013) showed that the *meta*-substituted phenylalanine prodrug of dopamine (Fig. 2W) was able to cross the BBB using LAT1, as the prodrug significantly inhibited the brain uptake of L-[^14^C]-leucine and its own brain uptake was reduced after co-perfusion with L-phenylalanine after *in situ* brain perfusion in rat (64). In contrast, the aspartic and adipic acid conjugates of dopamine (Fig. 2V,X) did not reach the brain as assessed with *in situ* brain perfusion in rat (64). However, in comparison to L-Dopa dosing in rats, the developed phenylalanine prodrug (Fig. 2W) did not increase the levels of dopamine in the striatum after i.p. injection (64). The L-tryptophan prodrug of benzoic acid (Fig. 2Y) has been designed based on the three-dimensional (3D) quantitative structure–activity relationship (QSAR) analysis of LAT1 binding of substrates by using classical and topomer comparative molecular field analysis and the data from the *in situ* rat brain perfusion technique (53). The prodrug showed the ability to inhibit the brain uptake of L-[^14^C]-leucine using *in situ* brain perfusion in rat (53).

The developed perforin inhibitor ester- (Fig. 3A) and amide-based (Fig. 3B) derivatives inhibited L-[^14^C]-leucine brain uptake after the *in situ* mouse brain perfusion (71). However, their brain uptake was not inhibited after co-perfusion with LAT1 substrate, L-tryptophan, suggesting utilization of transporter(s) other than LAT1. Interestingly, the cellular uptake of both derivatives was doubled by L-tryptophan in MCF-7 cells, providing additional evidence that the transporters other than LAT1 can be involved in their uptake (72). The cell uptake kinetics analysis, performed in MCF-7 cells, revealed involvement of two transporters, i.e. high-affinity low-capacity transporter and low-affinity high-capacity transporter (72). Interestingly, the uptake of ester-based prodrug of perforin inhibitor (Fig. 3A) into primary mouse neurons and immortalized microglia was reduced by approximately 25% by LAT1 inhibitor (KMH-233), while the uptake of this compound to mouse astrocytes decreased by around 70% by LAT1 inhibitor (74). Both prodrugs reached the brain after a single dose administered by i.p. injection in mice, while administration of the parent drugs did not result in BBB permeation. In addition, the parent drug was detected in the brain only after dosing with the ester-based prodrug (Fig. 3A) (71). Interestingly, the released parent drug was not detected in liver after a single dose i.p. injection of both prodrugs in mice. The ratio of liver AUC to plasma AUC for prodrugs (Fig. 3A, B) was significantly lower than that for parent drugs after a single dose i.p. injection in mice (71).

Recently, the structure-pharmacokinetics relationship of five derivatives of ketoprofen (Fig. 2N, Fig. 3C, D, E, F) designed to utilize LAT1 was investigated (57). The study confirmed the previous findings that the aromaticity presented in the prodrug’s promoiety plays a significant role in affinity to LAT1 and its utilization for cellular uptake of prodrugs. Thus, phenylalanine derivatives (Fig. 3C, D, E) but not derivatives with aliphatic promoieties, (Fig. 2N, Fig. 3F) significantly inhibited the uptake of L-[^14^C]-leucine in human retinal pigmented epithelial cells ARPE-19 cells. However, the uptake of all five derivatives was significantly inhibited by the LAT1 inhibitor, KMH-233 (66). Importantly, while all derivatives showed an ability to cross the mouse BBB according to *in situ* brain perfusion and could detected after a single i.p. injection of compounds in mice, the released parent drug was quantified in the brain only after administration of phenylalanine derivatives (Fig. 3C, D). The ratios of AUCs for the brain to plasma for unbound released ketoprofen from *meta-* and *para-*substituted phenylalanine derivatives (0.13 and 0.35, respectively) were significantly higher as compared to ketoprofen dosing (0.01) (66). In addition, five times higher ratios of AUCs for the brain to liver of ketoprofen released from these prodrugs were estimated as compared to ketoprofen i.p. dosing in mice (66). Thus, the study revealed that *meta-* or *para-*conjugation of phenylalanine directly to the parent drug molecule such as ketoprofen is an important structural feature which can provide targeted brain delivery of ketoprofen with reduced systemic exposure to the released parent drug. Importantly, an intra-brain distribution study of *meta-*conjugated phenylalanine prodrug of ketoprofen (Fig. 3C) using the brain slice method in mice and rats revealed a predominantly LAT1-mediated delivery of this prodrug to the intracellular compartment of the brain parenchyma in mice and rats (73). Thus, these findings suggest that in addition to reduced peripheral exposure to released parent drug, this prodrug enhanced the delivery of ketoprofen to the intracellular compartment of the brain parenchyma where the target, cyclooxygenase, is located. In addition, it has been demonstrated for the first time that the intra-brain distribution of LAT1-utilizing prodrug of ketoprofen (Fig. 3C) did not affect the transporter function such as amino acid transport and protein expression as shown in brain slices of mice and rats (73). In addition, Huttunen et al. (2019) reported that this prodrug accumulated in primary neurons and astrocytes, as well as in immortalized microglia *in vitro* (74). However, the role of LAT1 in the uptake of the prodrug to these cells was not confirmed. Moreover, the concentrations of the prodrug used in the study were higher than its *Km* value determined in the uptake studies in ARPE-19 cells (57) suggesting that at these concentrations the prodrug can utilize other transporter(s). Importantly, the findings of this structure-pharmacokinetic relationship analysis (57) and 3D QSAR (53) were confirmed in the study of derivatives developed to improve brain delivery of natural phenolic compound ferulic acid. The results revealed that the amide-based *meta-*conjugated phenylalanine derivatives of this agent (Fig. 2G, H) were efficiently bound to LAT1 and utilized the transporter for cellular uptake as well as permeating into the mouse brain after i.p. injection (56). However, the delivery of ferulic acid has not been improved after the dosing of these derivatives, as the AUC and C_max_ of the released ferulic acid in the brain after a single dose i.p. injection of *meta*-substituted phenylalanine derivative was more than three times lower than after parent drug administration (56).

In the studies of Puris et al. (2019) (56) and Huttunen et al. (2018) (75), the change of the linker from amide (Fig. 3C,G) to ester bond between the promoiety (Fig. 3I, J) and parent drugs (ferulic acid and ketoprofen, respectively), resulted in a loss of LAT1 substrate specificity *in vitro*. Therefore, the design of prodrugs/derivatives with either ester-or amide-linker needs to be justified for each parent drug on a case by case basis.

All in all, the results of these studies illustrate that LAT1 natural substrates might serve as a promising template for improving the delivery of drugs into the brain. However, the review revealed that only a few studies have demonstrated LAT1-mediated BBB permeation (61,62,64), i.e. for most of the compounds, it is only the affinity to LAT1 which has been investigated, either using *in situ* brain perfusion or *in vitro* cellular uptake inhibition experiments with LAT1 substrates. In addition, information about the intra-brain distribution has been provided only for two compounds (Fig. 2H, Fig. 3C). Moreover, there is a lack of the knowledge about the distribution to other tissues and therefore it is difficult to evaluate the brain targeting via LAT1, as only two studies (57,71) measured the distribution of (pro) drugs to liver and compared it to the brain distribution. Therefore, future studies should focus on more complex investigations of the delivery via LAT1 across the BBB, the cellular barrier of the brain parenchyma and other organs.

## Ocular delivery via LAT1

In conjunction with the improvement of the transport across the BBB, LAT1-mediated delivery has been used to circumvent poor permeability across the BRB. The LAT1 is expressed in the retina, where it is responsible for the exchange of amino acids (76,77) offering the opportunity for LAT1-mediated delivery of the drugs. Thus, several amino acid ester prodrugs of acyclovir have been developed, and the permeation of L-serine derivative of acyclovir (Fig. 3K) across cornea was inhibited by arginine and BCH, pointing to the involvement of cationic amino acid transporter 1 and LAT1 in its transport (78,79). Moreover, the derivative of acyclovir led to higher concentrations of acyclovir being released in the aqueous humour as compared to the parent drug in a study using a combination of the topical well infusion and aqueous humour microdialysis techniques in rabbits (79). Akanuma et al. (2018) demonstrated that [^3^H]-gabapentin utilized LAT1 for passage across the BRB in rats after carotid artery injection as well as cellular uptake *in vitro* in the models of the inner (TR-iBRB2 cells) and outer BRB (rat retinal pigment epithelial RPE-J cells), since the uptake of the compound was inhibited by LAT1 substrates (80). These studies hold promise that LAT1-mediated (pro) drug delivery could be used not only for the brain targeting, but also for the passage of the compounds across other LAT1 expressing barrier tissues such as BRB. However, additional studies will be required to investigate the efficiency of this strategy for the BRB delivery.

## Delivery of anticancer agents

Due to the significant up-regulation of LAT1 expression in several human tumors, LAT1 has been an attractive transporter for targeted delivery of amino acid-derived anticancer drugs and prodrugs as well as positron emission tomography (PET) probes for cancer diagnosis. In this chapter, we will summarize the studies focusing on the development of LAT1-utilizing compounds targeting cancer cells.

### Delivery of anticancer (pro)drugs

**Fig. 4:**
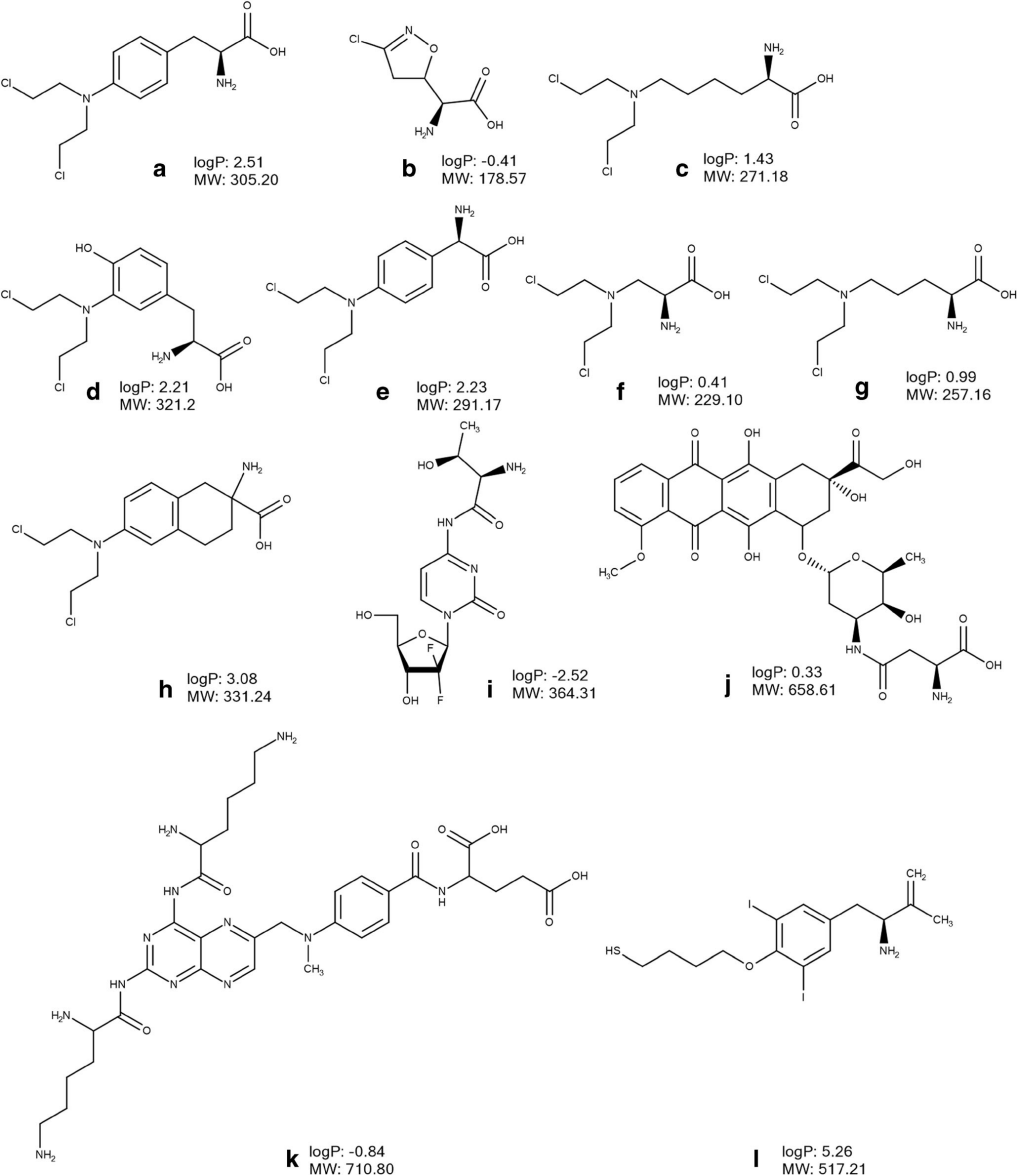
Chemical structures, molecular weight (MW) and logP of amino acid anticancer drugs and derivatives designed to utilize LAT1: melphalan (**a**), acivicin (**b**), derivatives of mustards (**c**-**g**), D,L-2-amino-7-bis[(2-chloroethyl)amino]-l,2,3,4-tetrahydro-2-naphthoic acid (**h**), derivative of gemcitabine (**i**), derivative of doxorubicin (**j**) derivative of methotrexate (**k**), 3CDIT (**l**). The values of logP were calculated using Marvin Sketch version 15.8.31 (ChemAxon, Budapest, Hungary).

LAT1 has been shown to be involved in transport of anticancer drugs such as melphalan (Fig. 4A) and acivicin (Fig. 4B). The L-phenylalanine prodrug melphalan (Fig. 4A), a widely used anticancer drug for the treatment of ovarian cancer and multiple myeloma, inhibited the uptake of L-leucine into human MDA-MB-231 breast cancer cells (81), T24 human bladder carcinoma cells (82) and in hLAT1 expressing *Xenopus* oocytes (16) providing evidence of binding to LAT1. Melphalan at therapeutic doses reduced the total number of cells and the live-to-dead cell ratio in two esophageal adenocarcinoma cell lines, i.e. Bic-1 and Seg-1, and in the SV-40-immortalized esophageal squamous cell line, Het-1A (83). The sensitivity to the effects of melphalan decreased after simultaneous incubation with the competitive LAT1 inhibitor BCH, demonstrating LAT1-mediated cellular uptake of the drug in the above-mentioned cell lines. Moreover, the transporter mediated passage of melphalan across the rat BBB after *in situ* brain perfusion (84). As melphalan is used for anti-cancer chemotherapy in patients with retinoblastoma, Hosoya et al. (2008) investigated the LAT1 binding of melphalan and other synthetic amino acid-conjugated mustards in LAT1 expressing conditionally immortalized rat retinal endothelial TR-iBRB2 cells (85). The study showed that melphalan inhibited the uptake of [^3^H]-phenylalanine, evidence in support of its binding to LAT1 (85). Furthermore, Hosoya et al. (2008) demonstrated that while lysine-mustard (Fig. 4C) and aromatic amino acid-mustards (Fig. 4D, E) significantly inhibited [^3^H]-phenylalanine uptake in TR-iBRB2 cells, aliphatic amino acid-mustards, such as alanine-mustard (Fig. 4F) and ornithine-mustard (Fig. 4G), did not bind to LAT1 (85). Interestingly, the analogue of melphalan, a nitrogen mustard amino acid D,L-2-amino-7-bis[(2-chloroethyl)amino]-l,2,3,4-tetrahydro-2-naphthoic acid (D,L-NAM) (Fig. 4H), utilized LAT1 for cellular uptake into murine L1210 lymphocytic leukaemia cells (86) and exhibited 50-times higher affinity for LAT1 at the rat BBB than melphalan after *in situ* brain perfusion (87). As a result, the BBB penetration of D,L-NAM in rats was more than 20 times greater than that of melphalan after *in situ* brain perfusion (87). In another study, several isomers (C-6 and C-8) of D,L-NAM (C-7 isomer) were synthetized in order to improve the affinity of the agent (88). However, when investigated using *in situ* rat brain perfusion, it was found that all compounds had lower affinity to LAT1 as compared to D,L-NAM.

LAT1 is involved in transport of another anticancer drug, acivicin (Fig. 4B), as demonstrated in a *trans*-stimulation assay of L-leucine efflux in human embryonic kidney HEK-LAT1 cells (23). However, the drug has failed due to unacceptable CNS toxicity caused by the high distribution of the compound to the brain. In cats, the toxic CNS effects could be reduced after concomitant administration of acivicin and a mixture of four large neutral amino acids or Aminosyn (mixture of 16 amino acids), i.e. evidence for the involvement of LAT1 in the brain distribution of the drug (89).

**Fig. 5:**
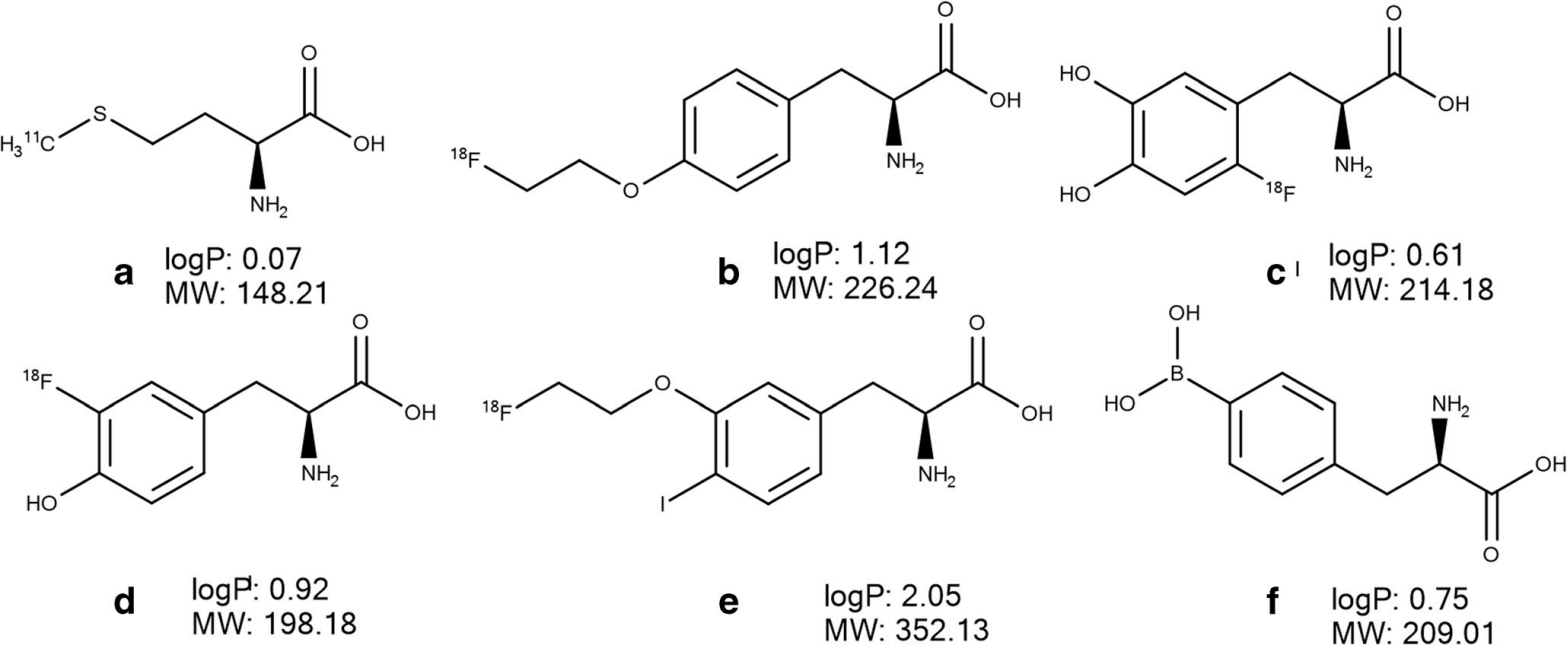
Chemical structures, molecular weight (MW) and logP of amino acid-based PET tracers L-[^11^C]-methyl-methionine (A), *O*-(2-[^18^F]-fluoroethyl)-L-tyrosine (B), 6-[^18^F]-fluoro-L-3,4-dihydroxy-phenylalanine (C), L-3-[^18^F]-fluoro-α-methyl tyrosine (D), (*S*)-2-amino-3-[3-(2-[^18^F]-fluoroethoxy)-4-iodophenyl]-2-methylpropanoic acid (E) as well as *p*-borono-phenylalanine (F). The values of logP were calculated using Marvin Sketch version 15.8.31 (ChemAxon, Budapest, Hungary).

Another attempt to apply an LAT1-mediated delivery approach for cancer targeting has been conducted by Hong et al. (2018) who developed a threonine-derivative of gemcitabine (Fig. 5I) (90). The compound evoked a greater cytotoxic effect in LAT1-overexpressing human pancreatic cancer cells, BxPC-3 and MIAPaCa-2, as compared to the parent drug (89). However, no evidence of LAT1-utilization by the derivative was reported; this compound does not fulfil the structural requirements of a LAT1 substrate. In another study, an aspartate derivative of doxorubicin (Fig. 4J) inhibited the uptake of L-[^3^H]-leucine in the S2-LAT1 transgene cell line demonstrating binding to the transporter (91). Importantly, in human liver cancer HepG2 (LAT1-positive) tumor-bearing mice, this derivative exerted a significantly stronger inhibition of tumour growth as compared to doxorubicin treatment (91). Singh et al. (2016) applied the LAT1-mediated prodrug approach to improve delivery of methotrexate to a brain tumour by conjugating the drug to lysine (Fig. 4K) via a carboxylic acid group (92). The compound demonstrated a four times greater distribution of released methotrexate between brain and plasma in comparison to that encountered after the i.v. administration of the parent drug in mice (92). However, the involvement of LAT1 in delivery of the conjugate was not investigated. Moreover, a biodistribution study in mice with i.v. administration of both the stable radiolabelled parent drug and conjugate showed that both compounds were widely distributed to many tissues including liver, spleen, lung and kidney (92).

There are several studies describing the potential of LAT1 utilization for enhanced delivery of nanoparticles and liposomes into cancer cells. Thus, An et al. (2016) intercalated doxorubicin into the adenosine-5′-triphosphate (ATP) responsive DNA scaffold, which was subsequently condensed and protected with a glutathione responsive polymer pOEI and functionalized with the LAT1 substrate 3CDIT (Fig. 4L) responsible for targeting the drug to tumour cells (93). The 3CDIT-targeting pOEI/doxorubicin/ATP aptamer nanoparticles demonstrated an outstanding accumulation in human-derived malignant glioma cells U87. Moreover, there was enhanced accumulation and doxorubicin release at the glioma tumour site as well as an improved antitumor therapeutic effect after i.v. injection of fluorescent-labelled nanoparticles in glioma model nude mice without any signs of systemic toxicity (93). In another study, Bhunia et al. (2017) developed LAT1 a selective liposomal drug carrier prepared from a novel L-Dopa functionalized amphiphile (Amphi-Dopa) (94). In the biodistribution study, the NIR-dye labelled liposomes of Amphi-Dopa were detected in the brain, liver and spleen at 4 h after i.v. administration but predominantly in the brain at 24 h in glioblastoma-bearing mice (94). The i.v. administration of the liposomes encapsulated with WP-1066, a potent signal transducer and activator of transcription 3 inhibitor, increased the overall survivability of mice bearing orthotopically established mouse glioblastoma dramatically (by ∼60%) as compared to that for the untreated mouse group (94).

Conjugation of liposomes and nanoparticles to glutamate has been used for improving the delivery of paclitaxel (95) and docetaxel (96) to cancer cells. The uptake of the liposomes in LAT1-expressing glioma cells was inhibited by leucine and phenylalanine only by 20–25% (96). The uptake of the nanoparticles was higher in LAT1-overexpressing cells (human cervix epitheloid carcinoma HeLa cells and MCF-7 cells) when compared to the situation in a LAT1 non-expressing cell line from Mouse Swiss NIH embryo NIH 3 T3 cells (95). Although based on these findings, the authors suggested LAT1-mediated delivery of the developed liposomes and nanoparticles, there is insufficient evidence for the involvement of LAT1 in the delivery of glutamine conjugates. In another study, the conjugation of L- and D-Dopa conjugated anisotropic gold nanoparticles increased the accumulation in several breast cancer cell lines (MCF-7, MDA-MB-231, MDA-MB-468, and MDA-MB-453) as compared to non-targeted nanoparticles conjugated with dopamine and 4-ethylcatechol (97). Moreover, the authors demonstrated LAT1-mediated accumulation of the L-Dopa functionalized anisotropic gold nanoparticles in MCF-7 cells, and the uptake was significantly inhibited by phenylalanine (97).

### Delivery of PET probes for cancer imaging

The knowledge of LAT1 overexpression in tumours and its substrate specificity has been exploited in the development of radiolabelled probes, transporter substrates, used in cancer diagnostics. The labelling of [^18^F] or [^11^C] in the LAT1 substrate structure allows PET imaging of accumulated compounds in cancer foci after their administration. Thus, several amino acids based probes have been developed including (*S*)-2-amino-3-[3-(2-[^18^F]-fluoroethoxy)-4-iodophenyl]-2-methylpropanoic acid ([^18^F]-FIMP), L-3-[^18^F]-fluoro-α-methyl tyrosine ([^18^F]-FAMT), 6-[^18^F]-fluoro-L-3,4-dihydroxy-phenylalanine ([^18^F]-DOPA), L-[^11^C]-methyl-methionine ([^11^C]-MET) and *O*-(2-[^18^F]-fluoroethyl)-L-tyrosine ([^18^F]-FET). [^11^C]-MET (Fig. 5A) has been the most commonly used radiolabelled amino acid due to its convenient and rapid synthesis (98) and high specificity in tumour detection, delineation and in the differentiation of benign from malignant lesions (99). In terms of LAT1-mediated delivery, Okubo et al. (2010) revealed that uptake of [^11^C]-MET in human newly diagnosed gliomas was correlated with the extent of LAT1 expression (100). Later, the development of another amino acid-based PET tracer [^18^F]-FET (Fig. 5B), a tyrosine analogue with a longer half-life of ^18^F, meant that it was possible to use this tracer in centres with no access to a cyclotron. The tracer provided information comparable to that obtained with [^11^C]-MET in the diagnostics of gliomas and brain metastases (101). Non-labelled FET induced approximately 1% efflux of preinjected phenylalanine in a *trans*-stimulation study in *Xenopus laevis* oocytes expressing h4F2hc-hLAT1 (102) indicative of low extracellular affinity to LAT1 or a slow transport rate of FET. In contrast, Habermeier et al. (2015) showed that extracellular FET stimulated the efflux of intracellular L-[^3^H]-leucine in *Xenopus laevis* oocytes expressing human h4F2hc-hLAT1, while preinjected FET to the oocytes did not stimulate L-[^3^H]-leucine influx into the cells, interpreted to mean that FET was an influx, but not an efflux LAT1 substrate (103). The discrepancy between the study findings of Lahoutte et al. (2004) and Habermeier et al. (2015) can be explained by the different transport kinetics of LAT1 substrates, phenylalanine and leucine, used in these studies (13). In addition, in the study of Habermeier et al. (2015), FET significantly inhibited the uptake of L-[^3^H]-tyrosine in LN229 glioblastoma cells suggesting that it was utilizing LAT1 as it accumulated in the oocytes expressing human h4F2hc-hLAT1, but not in control non-expressing LAT1 oocytes (103). The PET tracer [^18^F]-DOPA (Fig. 5C) has been widely used for research in the field of neuroendocrine tumours and movement disorders; it has been demonstrated an efficacy comparable to [^11^C]-MET for detecting brain tumors (104). In another study, the compound exhibited the best performance for the diagnostic of recurrent medullary thyroid carcinoma as compared to [^11^C]-MET, 2-deoxy-2-[^18^F]-fluoro-d-glucose ([^18^F]-FDG), Ga-somatostatin analogues and 3-*O*-methyl-6-[^18^F]-fluoro-DOPA (105). The uptake of [^18^F]-DOPA in biopsy samples of patients with newly diagnosed astrocytoma correlated with LAT1 expression (106). Tomiyoshi et al. (1997) synthetized an amino acid probe, [^18^F]-FAMT (Fig. 5D), which significantly inhibited LAT1-mediated uptake of L-[^14^C]-leucine and stimulated the efflux of preloaded L-[^14^C]-leucine indicating binding to LAT1 (107). In addition, the compound utilized the transporter for uptake in mouse renal proximal tubule cell line S2 stably expressing hLAT1 (108). Moreover, the uptake of [^18^F]-FAMT in the tumour correlated with the LAT1 expression level in patients with non–small-cell lung cancer (109) and oral squamous cell carcinoma (110). Recently, Nozaki et al. (2019) designed and synthetized a phenylalanine based tracer, [^18^F]-FIMP (Fig. 5E), which demonstrated greater tumor targeted delivery in the subcutaneous LAT1-positive human glioblastoma xenograft model as compared to [^18^F]-FET, [^11^C]-MET and [^18^F]-FDG (111).

### Boron neutron capture therapy

Targeting of anticancer agents via LAT1 has been applied for boron neutron capture therapy (BNCT) with the focus on patients suffering from high grade glioma (112,113). BNCT is a radiotherapy which is based on a nuclear fission reaction that occurs when ^10^B is irradiated with low energy thermal neutron beams and high-energy alpha-particles (^4^He^2+^) and recoiling lithium (^7^Li) nuclei (114). The efficacy of the BNCT is dependent on ^10^B accumulation in cancer tissue, which can be improved via LAT1-mediated delivery. Thus, *p*-borono-phenylalanine (BPA, Fig. 5F), a boron compound widely used in BNCT, demonstrated higher affinity to LAT1 as compared to LAT2 and ATB^0,+^ in *Xenopus laevis* oocytes expressing human h4F2hc-hLAT1 (115). However, the authors assumed that at therapeutic doses, the compound would be able to utilize both LAT1 and ATB^0,+^ for its uptake into cancer tissue (115).

Overall, these studies indicate that it should be possible to develop LAT1 selective compounds to be delivered via this transporter to cancer tissue for use in chemotherapy and cancer diagnostics. In this respect, a more systematic investigation of LAT1-mediated delivery and also a deeper understanding distribution of the compounds to healthy tissues are vital for the evaluation of the usefulness of the approach.

## Opportunities and limitations of LAT1-mediated drug delivery

The LAT1- mediated delivery is a promising approach, which has found applications not only in improving delivery of drugs, but also for diagnostic purposes**.** However, as the present review demonstrated, although a great number of (pro) drugs have been developed to improve targeted delivery via LAT1, only a limited number of LAT1-utilizing agents have entered clinical trials or are currently being used in clinical practice. In addition, only a few studies have demonstrated the utilization of LAT1 for delivery of investigated compounds; most of the studies lack evidence of LAT1-mediated uptake of the (pro) drugs or agents, which complicates the evaluation of the effectiveness of this approach. Furthermore, as there is no standardized procedure(s) for the evaluation of transporter-mediated delivery, the use of different methods (*in vitro*, *in situ* and *in vivo*) in the studies makes it difficult to compare the results such as transport kinetics parameters and distribution to target tissue.

There are several limitations of the approach which should be considered during the development of LAT1-utilizing (pro) drugs and agents. First, this delivery method is applicable for small-molecule drugs and not suitable for the delivery of macromolecules. The molecular weight of the summarized pro (drugs) with reported affinity to LAT1 and transporter-mediated uptake ranged between 148.21–513.56 g/mol. In the case of LAT1-mediated delivery of nanoparticles and liposomes, it seems evident that LAT1 plays a role in directing the nano-carrier to the cells expressing the transporter through the binding to LAT1 rather than delivering the nanoparticle via the transporter. Second, as discussed above (chapter “Brain delivery of CNS-acting drugs”), the LAT1-mediated (pro) drug approach provides predominant intracellular distribution of the substrates and therefore can benefit the delivery of the drugs with intracellular, but not extracellular targets. In addition, the affinity of (pro) drugs to LAT1 should be sufficient to be able to compete with natural substrates for LAT1 binding, but not enough to interfere with their homeostasis in the case of the non-cancerous tissue targeting. The review demonstrated that only limited information regarding the effect of LAT1-utilizing (pro) drugs on amino acid homeostasis is available; clearly future studies should address this issue.

Finally, one should remember that absolute targeting via LAT1 to a particular organ cannot be achieved due to the expression of LAT1 in several tissues and its overlapping substrate specificity with other transporters. The present review revealed that only a few studies have addressed the issue of targeting via LAT1 and investigated the systemic distribution of the compounds. Therefore, future research should focus on investigating the distribution of LAT1-utilizing (pro) drugs and agents to other tissues, in particular for cancer targeting. Thus, the selection of LAT1-mediated drug delivery should be carefully considered for each compound taking into account the advantages of the approach and its limitations.

## Conclusions

LAT1-mediated drug delivery is a promising approach, which has demonstrated its effectiveness for the brain- and cancer-targeted delivery of several agents, and it might be potentially used for delivery to other LAT-expressing tissues. In addition, several promising compounds have been developed and demonstrated the ability to bind to LAT1 and reach the target tissue in animal models. However, this review revealed that there is a lack of systematic knowledge of the efficiency of LAT1-mediated delivery *in vivo*, the distribution of compounds to non-target tissues, the proof of pharmacodynamic efficacy in disease models and translation of the data to humans. Therefore, further studies will be required to shed light on these issues if there is to be effective utilization of this approach in the targeted delivery of therapeutic compounds.

## Abbreviations

*APP*: Amyloid precursor protein
ATB^0,+^: b(0,+)-Type amino acid transporter
ATP: Adenosine triphosphate
AUC: Area under the total plasma or tissue concentration–time curve
BBB: Blood-brain barrier
BCH: 2-amino-2-norbornanecarboxylic acid
BNCT: Boron neutron capture therapy
BPA: *p*-borono-phenylalanine
BRB: Blood-retinal barrier
C_max_: Maximum total concentration of the drug achieved in the plasma or tissue
CNS: Central nervous system
D,L-NAM: D,L-2-amino-7-bis[(2-chloroethyl)amino]-l,2,3,4-tetrahydro-2-naphthoic acid
DNA: Deoxyribonucleic acid
IC_50_: Concentration of an inhibitor at which the response (or binding) is reduced by half
IL: Interleukins
i.p.: Intraperitoneal route of administration
i.v.: Intravenous route of administration
*K*_m_: Constant of Michaelis-Menten kinetics
LAT: L-type amino acid transporter
LPS: Lipopolysaccharide
mRNA: Messenger ribonucleic acid
PET: Positron emission tomography
*PSEN1*: Presenilin 1
QSAR: Quantitative structure-activity relationship
SLC: Solute carrier transporters
SV-40: Simian virus 40
*V*_*max*_: Maximal transport rate
3D: Three-dimensional space
[^11^C]-MET: L-[^11^C]-methyl-methionine
[^18^F]-DOPA: 6-[^18^F]-fluoro-L-3,4-dihydroxy-phenylalanine
[^18^F]-FAMT: L-3-[^18^F]-fluoro-α-methyl tyrosine
[^18^F]-FET: *O*-(2-[^18^F]-fluoroethyl)-L-tyrosine
[^18^F]-FIMP: (*S*)-2-amino-3-[3-(2-[^18^F]-fluoroethoxy)-4-iodophenyl]-2-methylpropanoic acid
